# Adoption of the Website and Mobile App of a Preventive Health Program Across Neighborhoods With Different Socioeconomic Conditions in the Netherlands: Longitudinal Study

**DOI:** 10.2196/32112

**Published:** 2022-02-02

**Authors:** Elena Agachi, Tammo H A Bijmolt, Jochen O Mierau, Koert van Ittersum

**Affiliations:** 1 Department of Marketing Faculty of Economics and Business University of Groningen Groningen Netherlands; 2 Department of Economics, Econometrics & Finance Faculty of Economics and Business University of Groningen Groningen Netherlands

**Keywords:** eHealth, mHealth, mobile health, mobile app, internet, preventive health program, health disparities, NSES, program adoption, survival analysis

## Abstract

**Background:**

Socioeconomic disparities in the adoption of preventive health programs represent a well-known challenge, with programs delivered via the web serving as a potential solution. The preventive health program examined in this study is a large-scale, open-access web-based platform operating in the Netherlands, which aims to improve the health behaviors and wellness of its participants.

**Objective:**

This study aims to examine the differences in the adoption of the website and mobile app of a web-based preventive health program across socioeconomic groups.

**Methods:**

The 83,466 participants in this longitudinal, nonexperimental study were individuals who had signed up for the health program between July 2012 and September 2019. The rate of program adoption per delivery means was estimated using the Prentice, Williams, and Peterson Gap–Time model, with the measure of neighborhood socioeconomic status (NSES) used to distinguish between population segments with different socioeconomic characteristics. Registration to the health program was voluntary and free, and not within a controlled study setting, allowing the observation of the true rate of adoption.

**Results:**

The estimation results indicate that program adoption across socioeconomic groups varies depending on the program’s delivery means. For the website, higher NSES groups have a higher likelihood of program adoption compared with the lowest NSES group (hazard ratio 1.03, 95% CI 1.01-1.05). For the mobile app, the opposite holds: higher NSES groups have a lower likelihood of program adoption compared with the lowest NSES group (hazard ratio 0.94, 95% CI 0.91-0.97).

**Conclusions:**

Promoting preventive health programs using mobile apps can help to increase program adoption among the lowest socioeconomic segments. Given the increasing use of mobile phones among disadvantaged population groups, structuring future health interventions to include mobile apps as means of delivery can support the stride toward diminishing health disparities.

## Introduction

### Background

Noncommunicable diseases currently account for more than half of the global burden of disease, causing an ever-increasing proportion of premature deaths in both low- and high-income countries [1]. This occurrence is driven by preventable factors such as unhealthy diets, lack of physical activity, and tobacco and alcohol consumption [2,3]. Although noncommunicable diseases affect all segments of the population, the socially disadvantaged groups experience higher risk factors for these diseases [4,5] while remaining hard to reach through preventive health interventions [6].

Socioeconomic disparities in the adoption of preventive health programs are a well-known challenge [7,8], with programs delivered via the web being a potential solution. Web-based health programs show higher prospects in terms of behavior change ability and accessibility as compared with offline programs [9-11], having predominantly two means of delivery: website and mobile app. However, no clear understanding exists yet as to which (if any) of these delivery means is better able to promote increased inclusivity of all population segments [12,13], or on the contrary, leads to reinforcement, or even widening, of the existing disparities [14,15].

### Objective

The health program examined in this study is the SamenGezond (from Dutch: Healthy Together) platform. Originally introduced by the health insurance company Menzis, the health program is aimed at the general Dutch population, following the goals of improving the health behaviors and wellness of its participants. The SamenGezond program was originally offered in the form of a website (introduced in 2012) and subsequently expanded to also include a mobile app (in 2017). Currently, reaching approximately 1 million participants, the program offers a set of activities and coaching that support healthy nutrition, physical activity, and other health behaviors.

With an increasing proportion of health programs being delivered through the web [16], it is of added value to gain a better understanding of the potential differential impact that the delivery means can have on program adoption. Therefore, the objective of this study is to analyze whether the adoption of a preventive health program by especially the low socioeconomic segment differs between the website and the mobile app. Identifying whether either of these means of delivery can achieve a better adoption rate among the socially disadvantaged groups could allow for future refining of health policy tools, contributing toward alleviating existing health disparities.

## Methods

### Study Sample

The database analyzed in this study originated from the SamenGezond platform, a large-scale web-based health program delivered by a health insurance company to the general Dutch population. The health program was introduced in July 2012, initially through a website, and starting in October 2017, expanded to also include a mobile app. The website of the health program mainly comprises information and coaching related to wellness and healthy lifestyle. The mobile app is an extension of the website, introducing several additional features to the health program, such as the ability to record activities with GPS, interact with an internet-based coach, and set and complete health goals. All program participants initially used the website of the program, with a subsequent choice of enrolling for the mobile app or continuing the use of solely the website. Although the health program is offered by a health insurance company, its participants are not solely clients of this company but can also be insured elsewhere (health insurance participation being legally mandated in the Netherlands).

The health program’s aim is to improve the lifestyle and wellness of its participants by focusing on physical activity, healthy eating habits, social activity, mental health, good sleep habits, and minimized stress. The activities that are provided involve entering or recording physical activities, reading articles, setting goals, including friends in challenges, answering health questions, being assisted by an internet-based coach, and forming a daily *fit-score* based on the individual activities within the platform. The program also offers benefits in the form of accumulated points from participation in the various sections of the platform, which can be used to acquire specific products, vouchers for various services, gadgets, or charity contributions.

Enrollment to the health program was open and free, and all the participants involved in this study provided their voluntary and informed consent. Approval for this project was obtained from the institutional review board of the University of Groningen.

Data were collected between 2012 and 2019 and analyzed in 2020 and 2021 within a longitudinal, nonexperimental study design. This study design was used as it allows for the examination of the duration until adoption of the 2 components of the health program for a large group of participants. The analyzed data had a weekly frequency, covering 376 weeks and including 83,466 participants. All program participants were aged >18 years and were residents of the Netherlands; no additional exclusion criteria were applied. When selecting the participants for this study, out of the 838,500 individuals who enrolled in the health program at the time, 404,398 (48.23%) individuals who had logged into the health program at least once in the past 2 years were examined for eligibility. Owing to limitations in data transfer and storage, approximately 24.73% (100,000/404,398) of the eligible group were invited randomly to participate in this study, with 83.46% (83,466/100,000) of them having provided their consent for participation. It is not possible to compare the analyzed sample with the approached sample, as no data were available on the individuals who did not provide their consent for data sharing.

### Measures

The effectiveness of health programs is defined by their ability to contribute to disease prevention, which critically hinges on individuals adopting and using the program. However, a reason causing overall ambiguity related to the benefits of web-based health programs is the significant number of programs that have been unsuccessfully adopted by individuals [17]. In addition, a slow rate of adoption can serve as an early indicator of potential dropout [18] and, subsequently, program failure, making it paramount to gain a better understanding of the health program adoption process, to support program success.

Building on the diffusion of innovations literature [19], this study measured the adoption of the health program using the number of individuals who signed up for the program each week (weekly subscription rate), with the rate of adoption being defined as the speed at which the health program spreads among the target group. Given that the adoption decision of individuals varies between technologies [20], this study focused in particular on the comparison of the rate of adoption of the health program between the website and mobile app across socioeconomic groups.

Examining the rate of adoption by distinguishing between population groups allows for assessing whether there are differences in the reach of the health program depending on the means of delivery. Given that solely individual factors offer insufficient explanations of differences in health behaviors [21], the neighborhoods in which individuals live have emerged as contexts affecting both health behaviors [22] and health outcomes [23]. On the basis of the discussion by Duncan and Kawachi [24], neighborhood socioeconomic status (NSES) was used in this study to distinguish between population segments with different socioeconomic characteristics.

The NSES measure was created in this study using data on key indicators for each neighborhood in the Netherlands, provided by the Central Bureau of Statistics [25]. Following the methodology outlined in the study by Dekker et al [26], the NSES measure was calculated using nonlinear iterative partial least squares principal component analysis on the following characteristics, given on a postcode level: average income, average property value, subsidized renting, share of high-income households, share of owner-occupied properties, share of low-income households, share of the population receiving unemployment benefits, share of the population receiving disability benefits, and share of the population receiving short-term unemployment benefits. NSES quintiles were used in the analysis of this study based on the constructed NSES measure, with a lower NSES quintile corresponding to lower levels of socioeconomic conditions.

To control for individual characteristics of the program participants, gender and age were included in the analysis as additional covariates. Moreover, as it can be expected that marketing campaigns that support the health program influence the rate of program adoption, the analysis was augmented with indicators for marketing activities taking place in each observed week or in the preceding week (to control for a lagged impact of marketing). The marketing campaigns considered were radio, television, and web-based campaigns.

### Statistical Analysis

This study used survival modeling, which encompasses statistical procedures aimed at analyzing the time until an event occurs; the event of interest in this study was the adoption of the health program. The baseline population of individuals who could decide to adopt the health program was the general Dutch population, which was the target group of the health program (the marketing activities related to the health program took place in the Netherlands and were aimed at the Dutch population). Registration for the analyzed program was voluntary and free, which made it possible to examine the true rate of adoption, as in such a setup, individuals are free to decide by themselves when to enroll in the health program [27].

**Figure 1:**
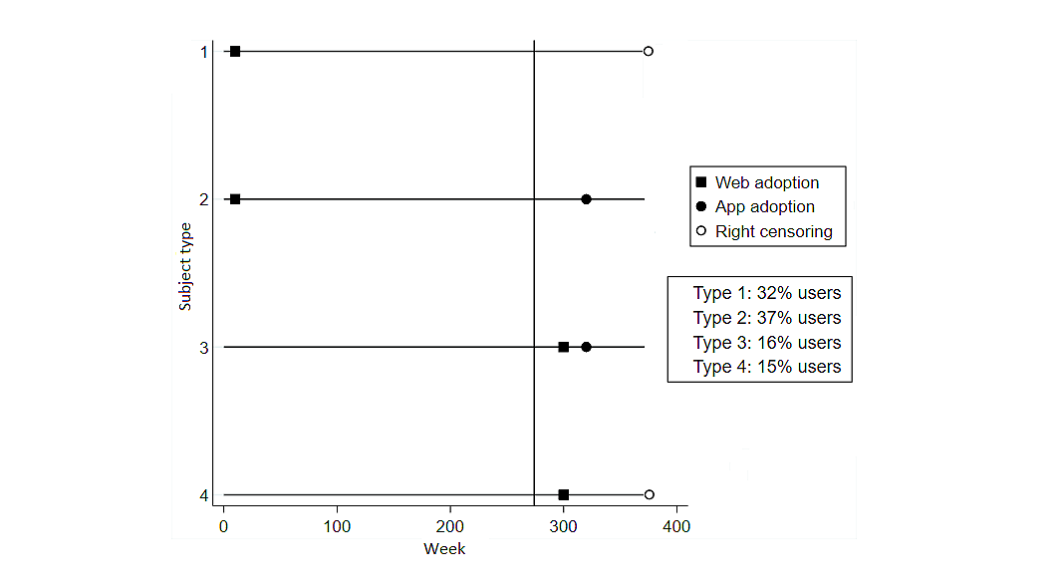
Types of health program participants.

All the observed participants of the health program could experience two events: the adoption of the health program through the website and the adoption of the mobile app, with the former always preceding the latter. This pattern is schematically reflected in Figure 1, which depicts the several types of health program participants possible, depending on whether and when a participant adopted the website and the mobile app of the health program.

Figure 1 summarizes the 4 types of health program participants. Type 1 includes users (26,908/83,466, 32.24% of the program users) who adopted the health program before the mobile app was introduced (in the period between 2012 and 2017) and subsequently did not adopt the app when it became available in October 2017. Type 2 includes the users (31,333/83,466, 37.54%) who adopted the program before the app introduction and subsequently also adopted the app. Type 3 includes the users (12,979/83,466, 15.55%) who adopted the program after the app introduction and subsequently also adopted the app. Type 4 includes the users (12,246/83,466, 14.67%) who adopted the program after the app introduction and subsequently did not adopt the app. As the data set included solely participants who adopted the health program before September 2019, the observations linked to participants who would adopt the program or its mobile app after 2019 are not available and were censored.

At the end of the observation period, of the analyzed participants, 46.91% (39,154/83,466) had not adopted the app (yet) and were using only the website to access the health program. Possible reasons for this occurrence could be unawareness about the existence of the app or unwillingness to use the app based on not needing the extended features offered by it. Alternatively, the reluctance toward the adoption of the mobile app can be linked to privacy concerns [28].

As reflected in Figure 1, the adoption of the website and the mobile app were sequential events, with each participant being at risk for only 1 of these events at a time. The modeling approach that handles this structure best is an extension of the classical Cox model [29], namely the variants of the Prentice, Williams, and Peterson (PWP) model [30-32]. To answer the research question of this study related to differences in the rate of adoption between means of delivery, the PWP Gap–Time (PWP-GT) model is most appropriate, which estimates the effects of the following event since the time from the previous event [30,32]. This is achieved using time-dependent strata, where the hazard function is allowed to vary from event to event [33]. The PWP model estimates unbiased effects [33] and provides SEs robust to within-subject correlation [34]. Statistical analysis was performed using the survival package [35,36] implemented within the R (R Foundation for Statistical Computing) environment for statistical computing [37].

Several robustness checks were performed for changes in the model specifications based on models accounting for nonproportional hazard rates, with the estimated parameters of the main PWP-GT model maintaining their direction and statistical significance (section 2 in Multimedia Appendix 1 [29,38,39], available on the web). Similarly, the models’ parameter estimates remained unchanged when estimating the PWP-GT model with solely the NSES quintiles as covariates when controlling for being insured at the company that had initially introduced the health program and when accounting for subsequent program use measured by the number of weekly log-ins (section 3 in Multimedia Appendix 1, available on the web). Additionally, following the STROBE (Strengthening the Reporting of Observational Studies in Epidemiology) recommendations, the checklist presented in Multimedia Appendix 2 has been completed.

## Results

### Characteristics of Participants

On average, the 83,466 participants who were analyzed were in the program for >3 years (mean number of weeks in the health program 186, SD 124 weeks) and aged between 18 and 80 (mean age 46.5, SD 15.5) years, and 56% (46,741/83,466) of them were female. Table 1 provides an overview of the study participants’ characteristics.

Among the participants’ characteristics outlined in Table 1 is their distribution across NSES quintiles, which showed a higher proportion of program participants in the lowest 2 NSES quintiles. The insurance company operates on a larger scale in areas with low socioeconomic conditions, and as most participants were clients of this insurance company, the overrepresentation of the lowest NSES quintiles was reflected in the participants’ distribution (a more detailed distribution of participants across NSES quintiles is discussed in section 1 in Multimedia Appendix 1, available on the web).

**Table 1:** Characteristics of study participants (N=83,466).

Key attributes		Values
Weeks covered, n		376
Number weeks in health program, mean (SD)		186 (124)
Participants using website alone, n (%)		39,146 (46.9)
Participants using website and mobile app, n (%)		44,320 (53.09)
Age (years), mean (SD)		46.5 (15.5)
Female participants, n (%)		46,741 (56)
Weeks with active marketing campaigns, n (%)		56 (14.9)
**Participants per age group (years), n (%)**		
	18-26	8263 (9.89)
	27-36	17,779 (21.3)
	37-46	16,693 (19.99)
	47-56	17,695 (21.2)
	57-66	12,937 (15.49)
	67-80	10,099 (12.09)
**Participants per NSES^a^ quintile (from lowest to highest socioeconomic conditions), n (%)**		
	First	18,446 (22.1)
	Second	22,453 (26.9)
	Third	16,109 (19.29)
	Fourth	13,605 (16.3)
	Fifth	12,853 (15.38)

^a^NSES: neighborhood socioeconomic status.

**Figure 2:**
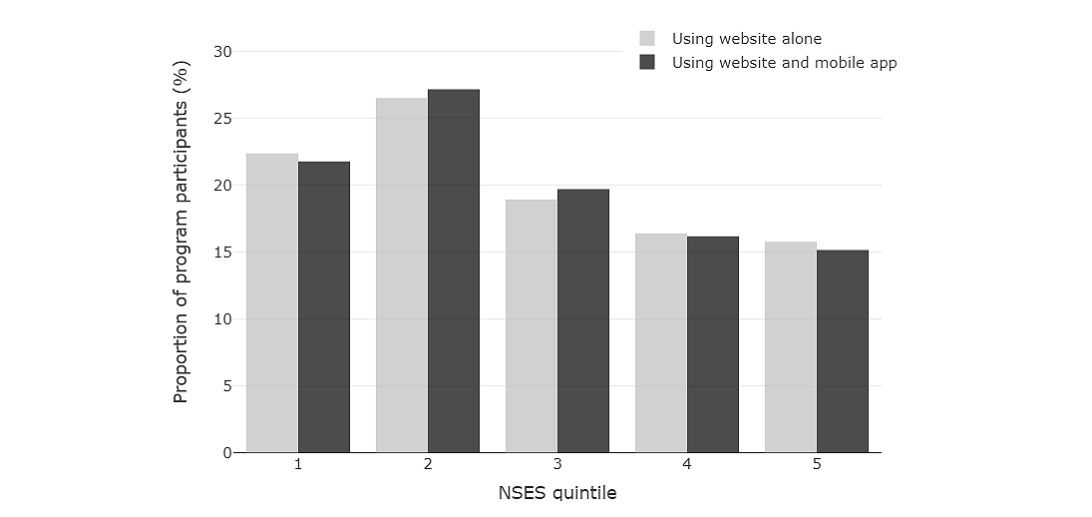
Distribution of health program participants across neighborhood socioeconomic status (NSES) quintiles.

Figure 2 further depicts the proportion of program participants who were in each NSES quintile, separated into two groups: those who used solely the website of the health program and those who used both the website and the mobile app.

On the basis of Figure 2, the distribution of program participants across NSES quintiles showed a similar pattern independent of the health program’s delivery means used.

### Regression Results

Analyzing the rate of adoption of the health program generated the results shown in Table 2, which contains the parameter estimates from the PWP-GT model accounting for NSES quintiles and the covariates of age, gender, and marketing indicators. The estimates shown in Table 2 are the hazard ratios (HRs; exponentiated model parameters) reflecting the effect size of the covariates, their corresponding 95% CIs, and *P* values.

**Table 2:** The impact of covariates on the rate of program adoption (Prentice, Williams, and Peterson Gap–Time model estimation results^a,b^).

Variables		Program adoption through website			Mobile app adoption	
		HR^c,d^ (95% CI)	*P* value	HR^d^ (95% CI)		*P* value
**NSES^e^ quintile**						
	First	1.000^f^	N/A^g^	1.000^f^		N/A
	Second	1.034 (1.015-1.054)	.002	0.940 (0.907-0.973)		.001
	Third	1.029 (1.008-1.051)	.02	0.954 (0.918-0.990)		.02
	Fourth	1.031 (1.009-1.053)	.02	0.950 (0.912-0.988)		.02
	Fifth	1.020 (0.997-1.043)	.12	0.948 (0.910-0.987)		.02
Age (in years)		1.007 (1.006-1.007)	<.001	0.980 (0.979-0.981)		<.001
**Gender**						
	Female	1.000	N/A	1.000		N/A
	Male	1.074 (1.060-1.088)	<.001	0.821 (0.797-0.845)		<.001
**Marketing**						
	No	1.000	N/A	1.000		N/A
	Yes	0.378 (0.360-0.396)	<.001	17.007 (16.979-17.035)		<.001

^a^Interpreting the estimated hazard ratios, for example, the second neighborhood socioeconomic status (NSES) quintile had an increased likelihood of program adoption via the website by a factor of 1.034 (95% CI 1.015-1.054) as compared with the lowest NSES quintile, keeping other covariates constant (equivalent to a 3.4% increased likelihood of adoption). On the other hand, the likelihood of adoption of the mobile app when comparing the second NSES quintile with the first one shows a decreased likelihood of adoption for the second NSES quintile by a factor of 0.940 (95% CI 0.907-0.973) or 6%.

^b^Observations=166,932 (the 166,932 observations reflect the 83,466 participants as the model accounts for 2 events per participant); R^2^=0.255; maximum possible R^2^=1.000; Wald test (*df*)=56,343.96 (14); *P*<.001.

^c^HR: hazard ratio.

^d^An HR of 1.000 was assigned to the reference level for each categorical covariate.

^e^NSES: neighborhood socioeconomic status.

^f^For the HR of 1.000 there is no 95% CI reported, as this is not an estimated HR, but is the default value assigned to the reference level.

^g^N/A: not applicable (it is the reference level).

The estimation results indicate that the impact of association with an NSES quintile on the rate of adoption of the health program differs between the 2 means of delivery. For the health program adoption through the website (shown in the first column of Table 2), most NSES quintiles have a statistically significant higher likelihood of adoption compared with the lowest NSES quintile; for example, an individual associated with the second NSES quintile has an increased likelihood of adoption by a factor of 1.034 (95% CI 1.015-1.054) compared with the lowest NSES quintile, keeping other covariates constant. However, for the adoption of the mobile app of the health program, all NSES quintiles have a lower likelihood of adoption compared with the lowest NSES quintile (shown in the second column of Table 2); comparing the second NSES quintile to the lowest one reveals a lower likelihood of adoption for the mobile app by a factor of 0.940 (95% CI 0.907-0.973), keeping other covariates constant. In addition, the estimated decrease in the likelihood of mobile app adoption for the higher NSES quintiles compared with the lowest one is higher than the estimated increased likelihood of website adoption (the effect sizes vary between a decreased likelihood of 4.6%-6% for the mobile app adoption and an increased likelihood of approximately 3% for the website adoption).

Examining the additional covariates shown in Table 2 indicate that older individuals have a higher rate of adoption of the website (HR=1.007, 95% CI 1.006-1.007) but a lower rate of adoption of the mobile app (HR=0.980, 95% CI 0.979-0.981) as compared with younger individuals. In addition, men are faster adopters of the website (HR=1.074, 95% CI 1.060-1.088) but slower adopters of the mobile app (HR=0.821, 95% CI 0.797-0.845) as compared with women. Finally, the weeks in which the marketing campaigns took place showed an increased rate of adoption for the mobile app (HR=17.007, 95% CI 16.979-17.035) but a decreased rate of adoption for the website (HR=0.378, 95% CI 0.360-0.396). The latter effect can be explained by the fact that all marketing campaigns took place after the introduction of the mobile app, when the website rate of adoption had already slowed down. Focusing on the impact of marketing campaigns on the rate of adoption of the mobile app based on interaction terms (section 3 in Multimedia Appendix 1, available on the web), it turns out that higher NSES quintiles are more sensitive to the marketing campaigns than the lowest NSES quintile.

## Discussion

### Principal Findings

Owing to the increasing need for the prevention of risk behaviors such as poor nutrition habits and insufficient physical activity, web-based health programs have emerged as sustainable means of providing large-scale preventive health services to the population. As lower socioeconomic segments frequently exhibit lower uptake levels of preventive health services, additional research is needed to identify whether the lower socioeconomic segments are more receptive to *web-based* health programs. In this study, we examine whether the website or mobile app delivery means of a web-based preventive health program can induce a higher likelihood of adoption among the population group with the lowest socioeconomic conditions.

Analyzing the distribution of health program participants across NSES quintiles revealed a higher proportion in the lowest 2 NSES quintiles. Although generally, higher socioeconomic segments tend to be more represented in preventive health programs [40], the overrepresentation of the lowest NSES quintiles observed in this study is linked to the particularities of the insurance company that introduced the program, which operates on a larger scale in areas with low socioeconomic conditions.

The main findings of this study show that the website of the health program is associated with a higher likelihood of adoption among the higher socioeconomic population groups (between 2% and 3% increased likelihood of adoption; *P* value between .12 and .002 depending on the NSES quintile), whereas the mobile app displays a higher likelihood of adoption among the lowest socioeconomic group (between 2% and 6% increased likelihood of adoption; *P* value between .02 and .001 depending on the NSES quintile). Additional findings originating from this study reveal that the individuals’ demographic characteristics are also linked to differences in adoption per means of delivery, with younger women (*P*<.001) more likely to adopt the mobile app of the health program. Marketing campaigns are estimated to increase the likelihood of mobile app adoption: 170% (*P*<.001) increased likelihood of mobile app adoption during, or right after, the weeks in which the marketing campaigns about the health program took place.

### Comparison With Previous Work

The findings in other existing research differ on the topic of health program adoption through mobile apps among socioeconomic groups. On the one hand, it is estimated that higher NSES segments are more likely to use health programs delivered through mobile apps [12,15] because of their possession of better digital skills [41] and easier access to technological devices [42]. On the other hand, when engaging with web-based health programs, individuals living in lower NSES areas do so mostly through mobile apps [13,43] while showing similar ease of use of mobile apps for health as that of groups with higher socioeconomic conditions [44,45].

For the findings in this study, a circumstance likely linked to the lower socioeconomic group showing a higher likelihood of mobile app adoption is the possession of digital skills. A characteristic of the Dutch population is the high levels of digital skills, with the Netherlands ranking highest in Europe on this scale [46]. An overall high level of digital proficiency removes the potential barriers that could prevent lower socioeconomic segments from engaging with health programs delivered through mobile apps. In addition, among communities with the lowest socioeconomic conditions in the Netherlands, there is a positive attitude toward web-based lifestyle programs [47]; this, combined with the high digital skills, potentially facilitates the adoption of the mobile app of the health program.

Overall, the topic of disparities in the use of preventive health services in the existing literature is supported by a general consensus that although the socially disadvantaged segments experience a heavier burden of behavioral risk factors and disease [48,49], they are generally the least represented group in preventive health services [40], an occurrence leading to an accelerating inverse social gradient [50]. With web-based health programs having the ability to achieve higher adoption and use rates [9-11], more research is warranted on the effects of specific delivery means of such programs on uptake, especially among the lower socioeconomic population groups.

The realization that mobile app delivery of preventive health programs can increase adoption among the lowest socioeconomic segment of the population has important implications for the future design of health programs. The current digital age is characterized by a higher prevalence of mobile phone use as compared with computer use, a pattern that is especially heightened in low socioeconomic groups [51,52], with mobile phone ownership and use also seeing a sharp increase in the low-income countries [53]. Given these tendencies, structuring future health programs to include a mobile app as a means of delivery can help to increase the adoption of such services among the disadvantaged socioeconomic segment, which can support the stride toward achieving health equity among all population groups.

In light of the growing health care expenditures and the associated health disparities, it is of importance for health insurance policies to encourage prevention over treatment. Given the higher burden of costs associated with the population segment with the lowest socioeconomic conditions, it is paramount to increase preventive health service use within this segment. Designing future health programs, including the use of mobile apps, can facilitate the increase in the use of such services by the lowest socioeconomic group, thus leading to cost savings and encouraging further investment toward large-scale, web-based prevention services.

### Limitations and Future Research

This study’s setup and analysis methods have several limitations.

First, as the adoption of the health program continued beyond the window of observation analyzed in this study, it is not confirmed that the effects observed here would maintain their validity when including the later adopters of the website and the mobile app. To verify whether the later adopters match the pattern discussed in this study, the current analysis can be replicated at a later stage of the health program’s existence.

Second, as the data analyzed were retrospective, the analysis was limited to only a few covariates related to the study’s participants. To overcome this restriction, the measure of NSES was used to reflect socioeconomic conditions, with the limitation of missing information on residential moves and not accounting for heterogeneity within neighborhoods. An extension of the current analysis would be to include a measure of individual socioeconomic status and compare the inferences based on the individual-level measure to the ones obtained here based on the neighborhood-level measure.

Third, the program analyzed has a particular structure of adoption, with all participants initially adopting the website and subsequently having the choice to adopt the mobile app. Such a structure can potentially lead to conservative estimates, as in this setup, the mobile app adopters are already aware of the program’s existence, having engaged previously with the website. Overcoming this restriction can be achieved by allowing program participants to adopt solely the mobile app, this being a structure toward which the analyzed health program is currently migrating.

Fourth, the specific health program analyzed includes solely individuals aged >18 years and from a high-income country. Given that youths worldwide are increasingly using more web-based services, future analyses could include younger individuals and compare web-based health program adoption within a more heterogeneous sample of low- and high-income countries. Although the findings of this study are based on the participants of a specific health program, we believe that the inferences drawn can be applied to the contexts of other health technologies, mainly because of the size and diversity of the data analyzed.

Finally, it is important to realize that solely adopting a preventive health program does not contribute to improvements in health. Therefore, as a future extension of this study, the analysis can include the subsequent use of the health program, and its impact on health outcomes, while distinguishing between population groups with different socioeconomic conditions.

### Conclusions

In this study, a large-scale web-based preventive health program promoted in the Netherlands was analyzed, focusing on its rate of adoption among socioeconomic groups. The mobile app of the health program was identified as a delivery means linked to a higher likelihood of program adoption among the population group with the lowest socioeconomic conditions. This finding suggests that future preventive health interventions can benefit from web-based delivery through mobile apps, especially in the light of the increasing use of mobile phones among the disadvantaged population segments.

## Appendix

Multimedia Appendix 1 Additional estimation results.

Multimedia Appendix 2 STROBE (Strengthening the Reporting of Observational Studies in Epidemiology) checklist.

## Abbreviations

HR: hazard ratio
NSES: neighborhood socioeconomic status
PWP: Prentice, Williams, and Peterson
PWP-GT: Prentice, Williams, and Peterson Gap–Time
STROBE: Strengthening the Reporting of Observational Studies in Epidemiology
