# Supplementary material for: Adoption of the Website and Mobile App of a Preventive Health Program Across Neighborhoods With Different Socioeconomic Conditions in the Netherlands: Longitudinal Study
Source: JMIR Hum Factors. 2022 Feb 2;9(1):e32112. doi: 10.2196/32112 (PMC8851331; doi:10.2196/32112)
Supplement: Multimedia Appendix 1 [file humanfactors_v9i1e32112_app1.doc]

# Appendix

Adoption of the Website and Mobile App of a Preventive Health Program across Neighborhoods with Different Socioeconomic Conditions in the Netherlands: Longitudinal Study

### **Appendix Section 1: Distribution of program participants versus insurance clients across neighborhood socio-economic (NSES) quintiles**

Appendix Figure 1 shows that the clients of the insurance company that has provided the health program are overly present in the lowest two NSES quintile. This is since the insurance company operates on a larger scale in areas with low socioeconomic conditions. Appendix Figure 1 also shows the distribution of the health program participants across NSES quintiles, highlighting the differences in distribution between the program participants and the insurance company clients. This difference is further depicted in Appendix Figure 2, where it is visible that out of all the NSES quintiles examined, health participants from the lowest NSES quintile are least involved in the health program.

**
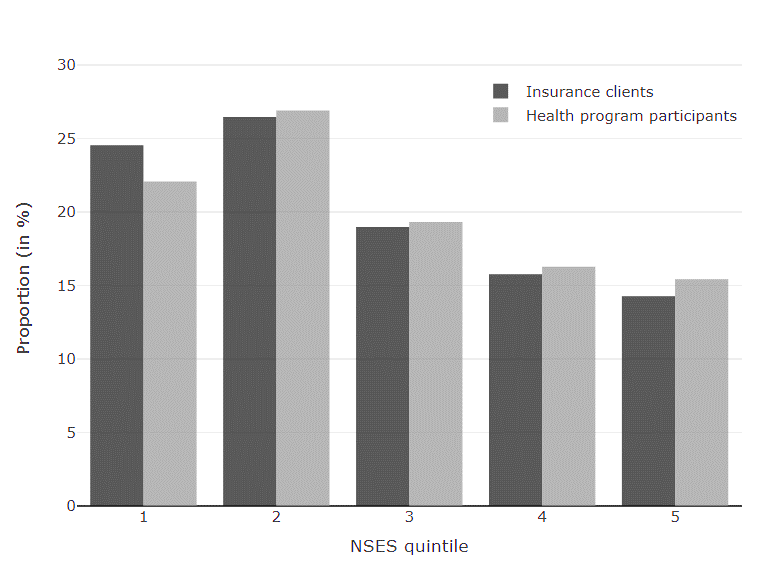
**

Appendix Figure 1: Comparison proportions of participants per NSES quintile between insured individuals and health program participants

**
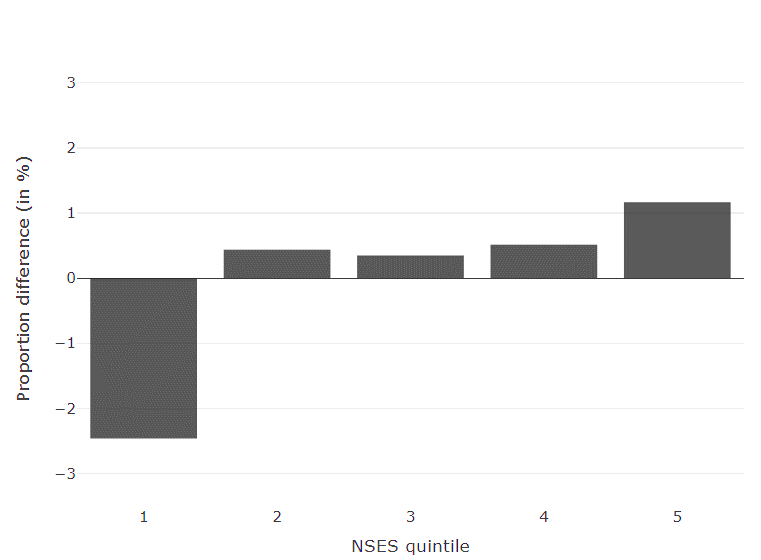
**

Appendix Figure 2: Difference in proportions of participants per NSES quintile between insured individuals and health program participants

### **Appendix Section 2: Non-proportional hazard**

The PWP-GT model employed for the analysis in this work, similar to the Cox regression models, operates under the proportional hazards assumption: the hazard ratios of individuals are independent over time[[1]](#footnote-2) (Cox and Oakes, 1984) [1]. The PWP-GT model employed, estimating the differences in rate of adoption for the website versus mobile app among NSES quintiles, does not fulfill the proportionality assumptions (based on the Schoenfeld (1982) residuals) [2]. The results of the test for proportionality of hazard are shown in Appendix Table 1, the null hypothesis for this test corresponding to a proportional hazard assumption being fulfilled.

Appendix Table 1: Estimation results for the test of proportionality of hazard based on the original PWP-GT model

| Variable | Chi-square value | Degrees of freedom | *P* value |
| --- | --- | --- | --- |
| NSES | 81.9 | 4 | <.001 |
| NSES:strata(EventNumber) | 24.8 | 4 | <.001 |

The results shown in Appendix Table 1 indicate that the proportionality of hazard assumption can not be accepted. In order to address the non-proportionality aspect, a different specification of the model is estimated, which uses time dependent coefficients *β*(*t*) in the PWP-GT model, based on step functions over periods of one year, following the approach of Therneau et al. (2017) [3]. This specification of the model fulfills the assumption of proportional hazard (Appendix Table 2) while leaving the parameter estimates of interest unchanged, suggesting that the initial estimates are robust to changes in the model that account for the non-proportional hazard at hand (estimation results shown in Appendix Table 3).

Appendix Table 2: Estimation results for the test of proportionality of hazard based on the adjusted PWP-GT model

| Variable | Chi-square value | Degrees of freedom | *P* value |
| --- | --- | --- | --- |
| NSES | 4.91 | 4 | .297 |
| NSES:strata(EventNumber) | 1.45 | 4 | .835 |
| NSES:strata(year) | 32.23 | 24 | .121 |

Appendix Table 3: PWP-GT model with time dependent coefficients

| Variables | Program adoption through website | | | | Mobile phone app adoption | | | |  | | | |
| --- | --- | --- | --- | --- | --- | --- | --- | --- | --- | --- | --- | --- |
|  | HR (95% CI)^a^ | | | *P* value | HR (95% CI)^a^ | | | *P* value | | |  | |
| NSES 2^nd^ quintile | 1.100 (1.065, 1.134) | | | <.001 | 0.929 (0.895, 0.964) | | | <.001 | | |  | |
| NSES 3^rd^ quintile | 1.169 (1.132, 1.206) | | | <.001 | 0.928 (0.891, 0.965) | | | <.001 | | |  | |
| NSES 4^th^ quintile | 1.175 (1.136, 1.213) | | | <.001 | 0.887 (0.848, 0.926) | | | <.001 | | |  | |
| NSES 5^th^ quintile | 1.190 (1.151, 1.229) | | | <.001 | 0.890 (0.850, 0.930) | | | <.001 | | |  | |
| Time strata covariates: | | HR (95% CI)^a^ | | | |  | *P* value | | | | | |
|  | |  |  | | |  |  | | | | | |
| NSES 2^nd^ quintile:strata(year 2) | |  | 0.973 (0.919, 1.027) | | |  | .311 | | | | | |
| NSES 3^rd^ quintile:strata(year 2) | |  | 0.929 (0.871, 0.987) | | |  | .012 | | | | |  |
| NSES 4^th^ quintile : strata(year 2) | |  | 0.957 (0.896, 1.017) | | |  | .146 | | | | | |
| NSES 5^th^ quintile : strata(year 2) | |  | 0.890 (0.828, 0.952) | | |  | <.001 | | | | | |
| NSES 2^nd^ quintile : strata(year 3) | |  | 0.976 (0.919, 1.033) | | |  | .436 | | | | | |
| NSES 3^rd^ quintile : strata(year 3) | |  | 0.870 (0.808, 0.932) | | |  | <.001 | | | | |  |
| NSES 4^th^ quintile : strata(year 3) | |  | 0.834 (0.768, 0.899) | | |  | <.001 | | | | | |
| NSES 5^th^ quintile : strata(year 3) | |  | 0.798 (0.731, 0.865) | | |  | <.001 | | | | | |
| NSES 2^nd^ quintile : strata(year 4) | |  | 1.016 (0.954, 1.077) | | |  | .658 | | | | | |
| NSES 3^rd^ quintile : strata(year 4) | |  | 0.903 (0.836, 0.970) | | |  | .008 | | | | |  |
| NSES 4^th^ quintile : strata(year 4) | |  | 0.896 (0.826, 0.967) | | |  | .007 | | | | | |
| NSES 5^th^ quintile : strata(year 4) | |  | 0.894 (0.823, 0.965) | | |  | .006 | | | | | |
| NSES 2^nd^ quintile : strata(year 5) | |  | 0.907 (0.851, 0.964) | | |  | .001 | | | | | |
| NSES 3^rd^ quintile : strata(year 5) | |  | 0.791 (0.730, 0.853) | | |  | <.001 | | | | |  |
| NSES 4^th^ quintile : strata(year 5) | |  | 0.793 (0.728, 0.858) | | |  | <.001 | | | | | |
| NSES 5^th^ quintile : strata(year 5) | |  | 0.794 (0.729, 0.860) | | |  | <.001 | | | | | |
| NSES 2^nd^ quintile : strata(year 6) | |  | 0.849 (0.791, 0.906) | | |  | <.001 | | | | | |
| NSES 3^rd^ quintile : strata(year 6) | |  | 0.772 (0.709, 0.834) | | |  | <.001 | | | | |  |
| NSES 4^th^ quintile : strata(year 6) | |  | 0.800 (0.735, 0.865) | | |  | <.001 | | | | | |
| NSES 5^th^ quintile : strata(year 6) | |  | 0.744 (0.677, 0.811) | | |  | <.001 | | | | | |
| NSES 2^nd^ quintile : strata(year 7) | |  | 0.921 (0.872, 0.969) | | |  | <.001 | | | | | |
| NSES 3^rd^ quintile : strata(year 7) | |  | 0.877 (0.825, 0.929) | | |  | <.001 | | | | |  |
| NSES 4^th^ quintile : strata(year 7) | |  | 0.873 (0.819, 0.928) | | |  | <.001 | | | | | |
| NSES 5^th^ quintile : strata(year 7) | |  | 0.861 (0.806, 0.916) | | |  | <.001 | | | | | |
|  | |  | | | | | | | |  | | |
| Observations | 647,234  0.0004 | | | | | | | | | |  | |
| R^2^ |  |  |  |  |  |  |  |  |  |  |  | |
| Max. Possible R^2^ | 0.984 | | | | | | | |  | | | |
| Wald Test | 283.390 (df = 32, *P* value <.001) | | | | | | | |  | | | |

^a^HR: hazard ratios; CI: confidence interval

### **Appendix Section 3: Alternative specifications of the main PWP-GT model**

Appendix Table 4: Estimated hazard ratios PWP-GT model (1) with only NSES quintiles and (2) with additional covariates

|  | | | | |
| --- | --- | --- | --- | --- |
|  | | | | |
|  |  | | | |
|  |  | | | |
|  |  | | | |
|  | Dependent variable: hazard rate | | | |
|  | (1) | | (2) | |
|  | HR (95% CI) | *P* value | HR (95% CI) | *P* value |
|  | | | | |
| NSESq2:Website | 1.048 (1.028, 1.067) | <.001 | 1.034 (1.015, 1.054) | .002 |
| NSESq3:Website | 1.049 (1.027, 1.070) | <.001 | 1.029 (1.008, 1.051) | .016 |
| NSESq4:Website | 1.055 (1.033, 1.077) | <.001 | 1.031 (1.009, 1.053) | .016 |
| NSESq5:Website | 1.042 (1.019, 1.064) | <.001 | 1.020 (0.997, 1.043) | .120 |
| NSESq2:App | 0.925 (0.892, 0.959) | <.001 | 0.940 (0.907, 0.973) | .001 |
| NSESq3:App | 0.917 (0.881, 0.953) | <.001 | 0.954 (0.918, 0.990) | .022 |
| NSESq4:App | 0.878 (0.840, 0.915) | <.001 | 0.950 (0.912, 0.988) | .017 |
| NSESq5:App | 0.885 (0.846, 0.923) | <.001 | 0.948 (0.910, 0.987) | <.001 |
| Age:Website |  | | 1.007 (1.006, 1.007) | <.001 |
| Male:Website |  | | 1.074 (1.060, 1.088) | <.001 |
| Marketing:Website |  | | 0.378 (0.360, 0.396) | <.001 |
| Age:App |  | | 0.980 (0.979, 0.981) | <.001 |
| Male:App |  | | 0.821 (0.797, 0.845) | <.001 |
| Marketing:App |  | | 17.007 (16.979,17.035) | <.001 |
|  | | | | |
| Observations | 166,932 | | 166,932 | |
| R^2^ | 0.0004 | | 0.255 | |
| Max. Possible R^2^ | 1.000 | | 1.000 | |
| Wald Test | 64.08 (df = 8,  *P* value <.001) | | 56,343.96 (df = 14,  *P* value <.001) | |
|  | | | | |

^a^HR: hazard ratios; CI: confidence interval

Appendix Table 4 shows a comparison between the parameter estimates from the model discussed in the main paper (column (2)) and parameter estimates from an alternative model, accounting solely for the NSES quintiles (column (1)). This comparison shows that also when not accounting for any additional covariates, the likelihood of adoption of the mobile app is lower for the higher quintiles as compared to the lowest one (an opposite effect to that observed for the adoption of the health program via website). Hence, the addition of extra covariates to the PWP-GT model does not influence the direction of the main effect observed.

Appendix Table 5 examines the link between program adoption and marketing campaigns per NSES quintile, by including interaction terms between NSES quintiles and the indicator for marketing campaigns. The estimation results show that for the adoption of the health program via website, the weeks in which marketing campaigns occurred are linked to lower likelihood of adoption. This effect can be explained by the fact that all marketing campaigns took place after the introduction of the mobile app, when the website rate of adoption had already slowed down. For adoption of the mobile app, the estimation results in Appendix Table 5 show that the weeks in which marketing campaigns have occurred are linked to higher likelihood of adoption, this effect having a stronger magnitude for higher NSES quintiles as compared to the lowest one.

Appendix Table 5: PWP-GT model with interaction terms for marketing indicators

| Variables | Program adoption through website | | Mobile app adoption | | | |
| --- | --- | --- | --- | --- | --- | --- |
|  | HR (95% CI)^a^ | *P* value | HR (95% CI)^a^ | *P* value | | |
| NSES 2^nd^ quintile | 1.067 (1.046, 1.089) | <.001 | 0.892 (0.845, 0.938) | <.001 | | |
| NSES 3^rd^ quintile | 1.067 (1.044, 1.091) | <.001 | 0.868 (0.818, 0.918) | <.001 | | |
| NSES 4^th^ quintile | 1.072 (1.047, 1.096) | <.001 | 0.848 (0.796, 0.901) | <.001 | | |
| NSES 5^th^ quintile | 1.067 (1.046, 1.089) | <.001 | 0.892 (0.845, 0.938) | <.001 | | |
| Marketing indicator | 0.401 (0.365, 0.438) | <.001 | 16.318 (16.262, 16.373) | | <.001 | |
| *Marketing:* |  |  |  |  | | |
| NSES 2^nd^ quintile:Marketing | 0.921 (0.871, 0.971) | <.001 | 1.075(1.001, 1.148) | .020 | | |
| NSES 3^rd^ quintile:Marketing | 0.924 (0.869, 0.978) | <.001 | 1.112 (1.032, 1.191) | .002 | | |
| NSES 4^th^ quintile:Marketing | 0.922 (0.865, 0.979) | <.001 | 1.139 (1.055, 1.223) | <.001 | | |
| NSES 5^th^ quintile:Marketing | 0.925 (0.867, 0.982) | <.001 | 1.172(1.087, 1.258) | <.001 | | |
| Observations | 166,932  0.241 | | | | | |
| R^2^ |  |  |  |  |  |  |
| Max. Possible R^2^ | 1.000 | | | | |  |
| Wald Test | 55,108.260 (df = 18, *P* value <.001) | | | | |  |

^a^HR: hazard ratios; CI: confidence interval

Appendix Table 6 examines whether the model estimates related to NSES differences in adoption of the two components of the health program maintain their validity when controlling for subsequent usage of the program. Column (1) shows the estimates from the basic PWP-GT model accounting for NSES quintiles. Column (2) includes as control variable the average number of weekly logins that each individual exhibited after adopting the program. Column (3) shows the model estimates based on a subset of participants that have accessed (logged-in to) the health program on average at least once a month in the period following their adoption of the health program. As shown in Appendix Table 6, controlling for subsequent program usage via weekly logins does not change the direction or power of the main effects observed (namely that the lowest NSES quintile is more likely to adopt the mobile app of the health program and less likely to adopt the website as compared to higher NSES quintiles).

Appendix Table 6: PWP-GT model (1) with only NSES quintiles, (2) including variable for weekly logins and (3) on a subset of participants who logged in at least once a month

|  | | | | | |  |  |
| --- | --- | --- | --- | --- | --- | --- | --- |
|  | | | | | |  |  |
|  | Dependent variable: hazard rate | | | | | | |
|  | (1) | | (2) | | (3) | | |
|  | HR  (95% CI) | *P* val. | HR  (95% CI) | *P* val. | | HR  (95% CI) | *P* val. |
|  | | | | | | |
| NSESq2:Website | 1.048  (1.028, 1.067) | <.001 | 1.055  (1.035, 1.076) | <.001 | | 1.040  (1.005, 1.074) | .022 |
| NSESq3:Website | 1.049  (1.027, 1.070) | <.001 | 1.077  (1.055, 1.100) | <.001 | | 1.058  (1.021, 1.095) | .002 |
| NSESq4:Website | 1.055  (1.033, 1.077) | <.001 | 1.077  (1.053, 1.101) | <.001 | | 1.033  (0.994, 1.072) | .009 |
| NSESq5:Website | 1.042  (1.019, 1.064) | <.001 | 1.061  (1.038, 1.085) | <.001 | | 1.026  (0.987, 1.066) | .175 |
| NSESq2:App | 0.925  (0.892, 0.959) | <.001 | 0.919  (0.885, 0.954) | <.001 | | 0.932  (0.879, 0.986) | .012 |
| NSESq3:App | 0.917  (0.881, 0.953) | <.001 | 0.891  (0.855, 0.928) | <.001 | | 0.918  (0.861, 0.974) | .004 |
| NSESq4:App | 0.878  (0.840, 0.915) | <.001 | 0.859  (0.855, 0.928) | <.001 | | 0.940  (0.880, 1.001) | .050 |
| NSESq5:App | 0.885  (0.846, 0.923) | <.001 | 0.869  (0.831, 0.908) | <.001 | | 0.989  (0.928, 1.050) | .730 |
| Logins:Website |  | | 0.742  (0.735, 0.749) | <.001 | |  |  |
| Logins:App |  | | 1.620  (1.593, 1.647) | <.001 | |  |  |
|  | | | | | | | |
| Observations | 166,932 | | 166,932 | | | 54,167 | |
| R^2^ | 0.0004 | | 0.091 | | | 0.0003 | |
| Max. Possible R^2^ | 1.000 | | 1.000 | | | 1.000 | |
| Wald Test | 64.08 (df = 8,  *P* value <.001) | | 4089 (df = 10,  *P* value <.001) | | | 16.600 (df=10,  P value<.001) | |
|  | | | | | | | |

^a^HR: hazard ratios; CI: confidence interval

Appendix Table 7 examines whether controlling for being insured has an impact on the observed effects. The parameter estimates in column (2) of Appendix Table 7 show that accounting for being insured does not change the main effect observed: that the lowest NSES segment has a higher likelihood of adoption for the mobile app and a lower likelihood of adoption for the website of the health program compared to higher NSES. Being insured is estimated to increase the likelihood of adoption of the program through the website, and decrease the likelihood of adoption of the mobile app.

Appendix Table 7: PWP-GT model (1) with only NSES quintiles, (2) accounting for being insured at the company who initially introduced the health program

|  | | | | |
| --- | --- | --- | --- | --- |
|  | | | | |
|  |  | | | |
|  |  | | | |
|  |  | | | |
|  | Dependent variable: hazard rate | | | |
|  | (1) | | (2) | |
|  | HR (95% CI) | *P* value | HR (95% CI) | *P* value |
|  | | | | |
| NSESq2:Website | 1.048 (1.028, 1.067) | <.001 | 1.048 (1.028, 1.068) | <.001 |
| NSESq3:Website | 1.049 (1.027, 1.070) | <.001 | 1.049 (1.028, 1.071) | <.001 |
| NSESq4:Website | 1.055 (1.033, 1.077) | <.001 | 1.055 (1.033, 1.078) | <.001 |
| NSESq5:Website | 1.042 (1.019, 1.064) | <.001 | 1.043 (1.021, 1.066) | <.001 |
| NSESq2:App | 0.925 (0.892, 0.959) | <.001 | 0.925 (0.892, 0.960) | <.001 |
| NSESq3:App | 0.917 (0.881, 0.953) | <.001 | 0.913 (0.877, 0.951) | <.001 |
| NSESq4:App | 0.878 (0.840, 0.915) | <.001 | 0.876 (0.840, 0.914) | <.001 |
| NSESq5:App | 0.885 (0.846, 0.923) | <.001 | 0.880 (0.843, 0.919) | <.001 |
| Insured:Website |  | | 1.727 (1.624, 1.837) | <.001 |
| Insured:App |  | | 0.167 (0.148, 0.188)_ | <.001 |
|  | | | | |
| Observations | 166,932 | | 166,932 | |
| R^2^ | 0.0004 | | 0.006 | |
| Max. Possible R^2^ | 1.000 | | 1.000 | |
| Wald Test | 64.08 (df = 8,  *P* value <.001) | | 918.9 (df = 10,  *P* value <.001) | |
|  | | | | |

^a^HR: hazard ratios; CI: confidence interval

**References**

1. Cox DR, Oakes D. *Analysis of survival data*. Vol. 21. CRC press; 1984.
2. Schoenfeld D. Partial residuals for the proportional hazards regression model. *Biometrika*. 1982;69(1):239-241. doi:10.1093/biomet/69.1.239.
3. Therneau T, Crowson C, Atkinson E. (2013). Using time dependent covariates and time dependent coefficients in the Cox model. The survival package*. R help guide*. 2018. [https://cran.microsoft.com/snapshot/2018-07-06/web/packages/survival/vignettes/timedep.pdf](about:blank). Accessed February 1, 2021.

1. If a user has a probability of adopting the health program that is twice as high as that of another user, then at all later times the probability of adoption remains twice as high. [↑](#footnote-ref-2)
